# Sequestration of nanoparticles by an EPS matrix reduces the particle-specific bactericidal activity

**DOI:** 10.1038/srep21379

**Published:** 2016-02-09

**Authors:** Qian Wang, Fuxing Kang, Yanzheng Gao, Xuewei Mao, Xiaojie Hu

**Affiliations:** 1 State Key Laboratory of Lake Science and Environment, Nanjing Institute of Geography and Limnology, Chinese Academy of Sciences, Jiangsu 210008, China; 2 Institute of Organic Contaminant Control and Soil Remediation/College of Resources and Environmental Sciences, Nanjing Agricultural University, Jiangsu 210095, China; 3 School of Earth Sciences and Engineering, Nanjing University, Jiangsu 210046, China

## Abstract

Most artificial nanomaterials are known to exhibit broad-spectrum bactericidal activity; however, the defence mechanisms that bacteria use based on extracellular polymeric substances (EPS) to detoxify nanoparticles (NPs) are not well known. We ruled out the possibility of ion-specific bactericidal activity by showing the lack of equivalent dissolved zinc and silicon toxicity and determined the particle-specific toxicity of ZnO and SiO_2_ nanoparticles (ZnONPs/SiO_2_NPs) through dialysis isolation experiments. Surprisingly, the manipulation of the *E. coli* EPS (i.e., no EPS manipulation or EPS removal by sonication/centrifugation) showed that their particle-specific bactericidal activity could be antagonized by NP-EPS sequestration. The survival rates of pristine *E. coli* (no EPS manipulation) reached 65% (ZnONPs, 500 mg L^−1^) and 79% (SiO_2_NPs, 500 mg L^−1^), whereas survival rates following EPS removal by sonication/centrifugation were 11% and 63%, respectively. Transmission electron microscopy (TEM) combined with fluorescence micro-titration analysis and Fourier-transform infrared spectroscopy (FTIR) showed that protein-like substances (N-H and C-N in amide II) and secondary carbonyl groups (C=O) in the carboxylic acids of EPS acted as important binding sites that were involved in NP sequestration. Accordingly, the amount and composition of EPS produced by bacteria have important implications for the bactericidal efficacy and potential environmental effects of NPs.

ZnO and SiO_2_ nanoparticles (ZnONPs and SiO_2_NPs) have been widely used in catalysis, optical devices, electronic applications, biosensors, and the pharmaceutical industry^1^. They will inevitably be introduced into the environment through the manufacture, use, disposal, and recycling of commercial products^2^,^3^,^4^. The prevalence and harmfulness of nanoparticles (NPs) makes it necessary to probe their potential toxic and biological effects on the environment.

Most NPs have broad-spectrum bactericidal activity; however, the major mechanism by which NPs exert toxicity on bacteria and other organisms is controversial. Particle-specific biological activity is considered to be the main bactericidal mechanism of NPs, as supported by some informative evidence^5^,^6^,^7^,^8^,^9^,^10^. Other researchers have reported that the bactericidal activity of NPs correlates well with ion-specific effects, which are dependent on the free hydrated metal ions or soluble labile metal complexes released by the NPs^11^,^12^. Nevertheless, free hydrated ions or soluble labile metal ions released by ZnONPs and SiO_2_NPs exhibit low ion toxicity due to their higher median lethal doses (LD_50_). Thus, they are inefficient and cannot competently control the growth of bacteria and other organisms compared to a leachate of Ag nanoparticles (AgNPs)^12^. For instance, the LD_50_ of zinc ions toward *E. coli* is 17.0 mg L^−1^^13^, which is substantially greater than that of silver ions (LD_50_ = 0.13 mg L^−1^ for Ag^+^)^14^. Overall, whether the low-oxidation-state ions released from ZnONPs and SiO_2_NPs are responsible for their bactericidal activity is debatable.

Bacteria can secrete and embed themselves in an apparently electronegative extracellular polymeric substance (EPS, ɛ = −14 ~ −38 mV^15^) matrix that is composed primarily of polysaccharides and proteins^16^,^17^. These constituents are amphiphilic in nature and contain multiple active functional groups and/or charged moieties (e.g., amino and carboxyl groups) as well as hydrophobic moieties^18^ that protect the bacterial cells against environmental stresses such as desiccation, toxic metal species, antibiotic agents, high salinity, extreme temperature, and pH conditions^19^. Recently, bacteria were reported to antagonize the bactericidal activity of Ag-loaded carbon nanotubes by the exertion of the EPS^20^ or to antagonize the Ag^+^ via an EPS reduction process^14^. Other researchers have suggested that these extracellular biomolecules can effectively “embellish” the NPs to impart them with ‘biological identity’, thereby altering the cell membrane affinity, uptake, and retention of NPs^21^ and reducing their bactericidal activity. These works suggest that the EPS matrix on the bacterial surface plays a protective role in reducing toxicity^22^; however, the underlying molecular mechanisms through which the bacteria secrete the EPS to detoxify NPs have not been fully elucidated. Given the abundance and ubiquity of EPS in aquatic environments^19^,^23^, its role in reducing the bactericidal activity of NPs warrants further investigation.

This study provides direct evidence that EPS can protect bacteria through sequestering nanoparticles, thereby reducing the particle-specific bactericidal activity of ZnONPs and SiO_2_NPs. First, a set of dialysis isolation experiments was performed to demonstrate that ZnONPs/SiO_2_NPs exhibit a definitive particle-specific toxicity; these experiments ruled out an ion-specific biological effect by demonstrating the lack of toxicity of an equivalent amount of dissolved zinc (<30 mg Zn L^−1^) and silicon (<5.5 mg Si L^−1^). Second, the manipulation of the EPS in *Escherichia coli* suspensions (e.g., pristine *E. coli* or the removal of the EPS attached to the cells by sonication/centrifugation) demonstrated its critical role in reducing the particle-specific bactericidal activity. Fourier-transform infrared spectroscopy (FTIR) and transmission electron microscopy (TEM) combined with fluorescence micro-titration were used to characterize the sequestration of ZnONPs and SiO_2_NPs and the changes in the EPS groups before and after reaction with ZnONPs/SiO_2_NPs. The underlying mechanisms were also discussed.

## Results and Discussion

### Negligible ion-specific bactericidal activity of ZnONPs/SiO_2_NPs

**Figure 1:**
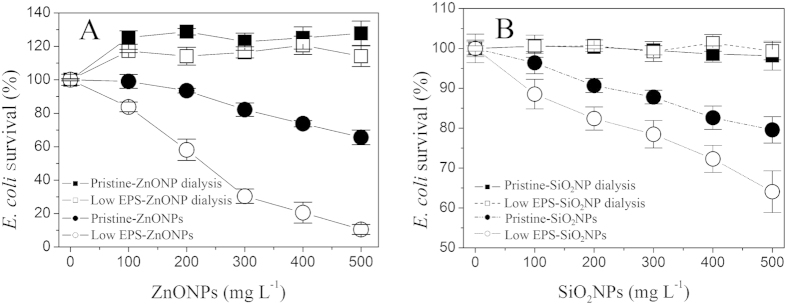
Effect of EPS on *E. coli* survival in the presence of various NP concentrations after 16 h of incubation. (**A**) ZnONPs; (**B**) SiO_2_NPs. The dose-response relationships were determined under four test conditions: pristine-ZnO/SiO_2_NP dialysis (◼) (pristine *E. coli* with NP dialysis); low EPS-ZnO/SiO_2_NP dialysis (◻) (EPS removal with NP dialysis); pristine-ZnO/SiO_2_NPs (●) (pristine *E. coli*); and low EPS-ZnO/SiO_2_NPs (○) (EPS removal by sonication/centrifugation). For all of the aforementioned NP dialysis treatments, the NPs were separated from *E. coli* by a dialysis membrane. In all tests, an inoculum of 1.3 × 10^7^ cell mL^−1^ was used. Error bars represent standard deviations of triplicate samples.

The particle-specific bactericidal activity of NPs toward *E. coli* was confirmed by a set of dialysis isolation experiments (Fig. 1A,B). In this device, particle-specific interactions do not occur because the NPs are enclosed inside a dialysis membrane, separated from the *E. coli* cells; however, the membrane allows soluble ions released by the aqueous NPs to penetrate into the LB medium containing *E. coli*. Figure 1A shows that, under the enclosed-ZnONP conditions (100–500 mg L^−1^), the survival rate of the pristine *E. coli* with no EPS manipulation (pristine-ZnONP dialysis) was approximately 128%, which is slightly higher than that of *E. coli* with the low EPS treatment (EPS removal by ultrasonic/centrifugation) of approximately 118% (low EPS-ZnONP dialysis). However, the survival rate was considerably higher than that of the *E. coli* without the dialysis treatment (pristine-ZnONPs and low EPS-ZnONPs). The survival rates of the *E. coli* with no EPS manipulation (pristine-ZnONP) and with low EPS (low EPS-ZnONP) were only 65% and 11%, respectively, in 500 mg L^−1^ ZnONPs. These results suggest that the decrease in *E. coli* survival is attributable to the particle-specific bactericidal effect of ZnONPs. This hypothesis was confirmed by the similar outcome for SiO_2_NPs (Fig. 1B). Notably, for the dialysis isolation experiment involving ZnONPs (pristine-ZnONP dialysis and low EPS-ZnONP dialysis), the *E. coli* survival rates were greater than 100% (Fig. 1A), suggesting that the small amount of soluble zinc released by the ZnONPs can promote *E. coli* survival as potential nutrients compared to the soluble silicon released by SiO_2_NPs (Fig. 1B).

**Figure 2:**
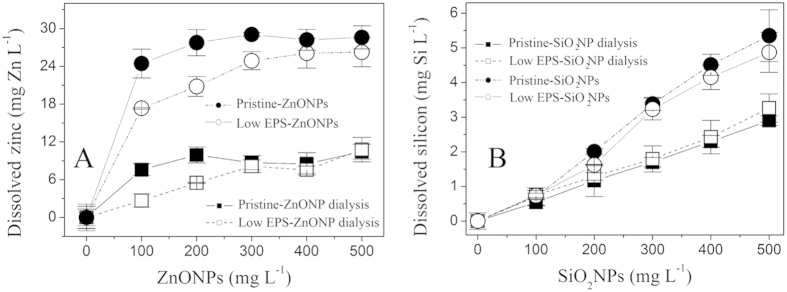
Dissolution curves of ZnONPs and SiO_2_NPs (50 mg L^−1^) in pristine-ZnO/SiO_2_NP dialysis (◼) (pristine *E. coli* with NP dialysis), low EPS-ZnO/SiO_2_NP dialysis (◻) (EPS removal with NP dialysis), pristine-ZnO/SiO_2_NPs (●) (pristine *E. coli*), and low EPS-ZnO/SiO_2_NPs (○) (EPS removal by sonication/centrifugation). (**A**) ZnONPs; (**B**) SiO_2_NPs. Dissolved zinc and silicon concentrations were determined after filtration through a 0.22-μm membrane. Error bars represent standard deviations of triplicate samples.

**Figure 3:**
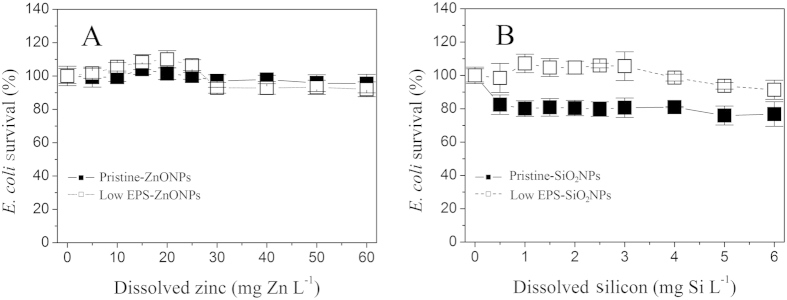
Effect of equivalent dissolved ion concentrations (Zn^2+^ and SiO_3_^2−^) on *E. coli* survival in the presence and absence of EPS after 16 h of incubation. (**A**) ZnONPs; (**B**) SiO_2_NPs. Pristine-ZnO/SiO_2_NPs (◼) (i.e., pristine *E. coli*), and low EPS-ZnO/SiO_2_NPs (◻) (EPS removal by sonication/centrifugation). In all tests, an inoculum of 1.3 × 10^7^ cell mL^−1^ was used. Error bars represent standard deviations of triplicate samples.

The ion-specific bactericidal activity of NPs was also ruled out by a set of NP dissolution experiments (Fig. 2A,B) and a set of equivalent ionic toxicity tests (Fig. 3C,D). Figure 2A,B show the corresponding dissolution curves of ZnONPs and SiO_2_NPs for each treatment according to the results in Fig. 1. The amount of ZnONP dissolution was gradually enhanced as the concentration of ZnONPs was increased to 500 mg L^−1^ (Fig. 2A). At a ZnONP concentration of 500 mg L^−1^, the extent to which the ZnONPs dissolved exhibited a descending order: pristine-ZnONPs (29 mg Zn L^−1^) > low EPS-ZnONPs (27 mg Zn L^−1^) ≫ pristine-ZnONP dialysis (10 mg Zn L^−1^) ≈ low EPS-ZnONP dialysis (10.2 mg Zn L^−1^). Similarly, the pristine *E. coli*-induced dissolution of SiO_2_NPs (pristine-SiO_2_NPs) was slightly higher than that induced by the low-EPS *E. coli* (Fig. 2B), and both were apparently higher than that of the pristine *E. coli* and low-EPS *E. coli* with SiO_2_NP dialysis. These results suggest that the EPS can solubilize the NPs upon coming into direct contact. The dissolution of NPs can be reconfirmed by particle size analysis (Figure S1A–D of the Supplementary Information). After reaction with *E. coli*, their diameters decrease from 15 nm to 11 nm for ZnONPs and 13 nm for SiO_2_NPs, respectively. Nevertheless, free ions released by the ZnONPs/SiO_2_NPs cannot kill *E. coli*. On the basis of the dissolution concentrations of the ZnONPs (<30 mg L^−1^ dissolved Zn) and SiO_2_NPs (<5.5 mg L^−1^ dissolved Si), equivalent ionic gradients were used to determine the effect of dissolution on *E. coli* survival in the presence and absence of the EPS after 16 h of incubation (Fig. 3A,B). The results indicate that, although the ZnONP concentration increased to 60 mg Zn L^−1^ (the maximal amount of ZnONP dissolution was less than 30 mg Zn L^−1^ in Fig. 2A), the *E. coli* survival rate still reached 95–110%, indicating that a dissolved zinc concentration of less than 60 mg L^−1^ does not inhibit the pristine and low-EPS *E. coli* survival. Similar results are shown in Fig. 3B, suggesting that the dissolved silicon negligibly affects the survival of pristine *E. coli* (pristine-SiO_2_NPs) when the concentration of dissolved silicon (SiO_3_^2^) is increased to 6 mg Si L^−1^. These results confirm that the released soluble zinc and silicon are not the main reason for the decrease in *E. coli* survival. The bactericidal activity of the ZnONPs/SiO_2_NPs is thus attributed to the particles themselves.

### EPS enhances *E. coli* survival under NP-exposure conditions

Figure 1 shows the survival rates of pristine *E. coli* with no EPS manipulation (●) (pristine *E. coli*) and with low EPS (○) (EPS removal by sonication/centrifugation). And it is easily to seen the EPS permeable barrier (thickness of 160–290 nm) of pristine *E. coli* with no EPS manipulation (Figure S2A and B of the Supplementary Information). The survival rates decrease with increasing ZnONP or SiO_2_NP concentration, reflecting the clear bactericidal effect on *E. coli*. Nevertheless, the bactericidal activity was significantly mitigated by the EPS adhering to the *E. coli* surface. At ZnONPs and SiO_2_NP concentrations as high as 500 mg L^−1^, the survival rate of the pristine *E. coli* reaches 65% (pristine-ZnONPs) and 79% (pristine-SiO_2_NPs), compared to corresponding values of 11% (low EPS-ZnONPs) and 63% (low EPS-SiO_2_NPs), respectively, in the presence of low EPS. Apparently, the survival of pristine *E. coli* (pristine-ZnONPs/SiO_2_NPs) in the presence of EPS is greater than that of low-EPS *E. coli* (low EPS-ZnONPs/SiO_2_NPs), suggesting that the EPS matrix protects the *E. coli* against particle-specific stress from the NPs.

**Figure 4:**
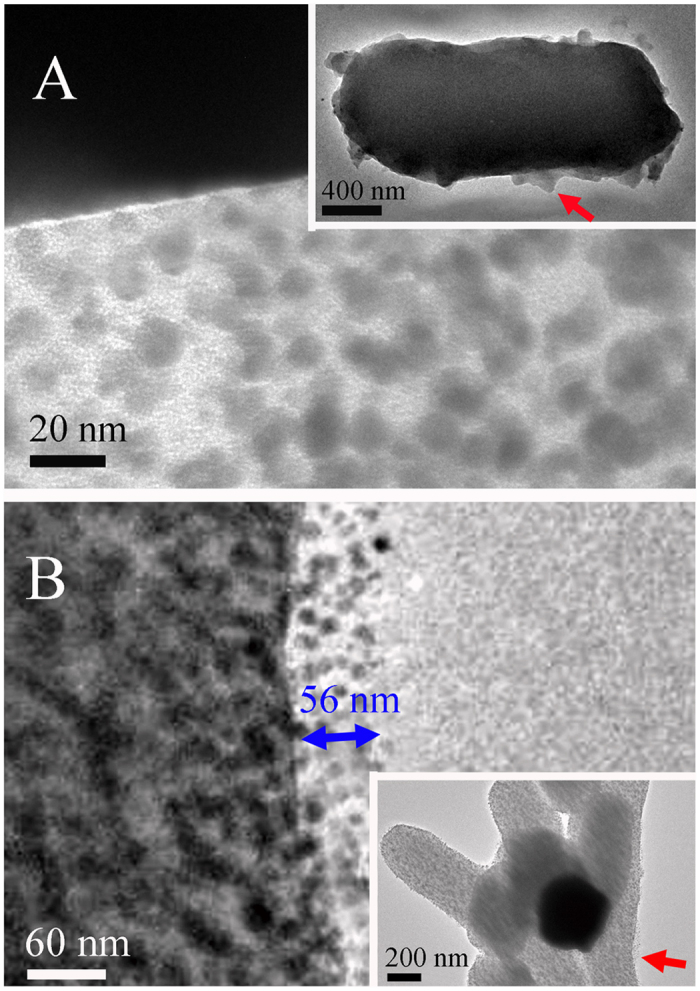
TEM images of ZnONPs (**A**)/SiO_2_NPs (**B**) entrapped within the EPS matrix on the surface of *E. coli* (1.3 × 10^7^ cell mL^−1^). Red arrows indicate the amplified sites on the surface of *E. coli*. Magnified images are shown in the bottom-left portion of (**A**) (ZnONPs) and top-left portion of (**B**) (SiO_2_NPs). The blue arrow indicates the average thickness of NP layer on on the surface of *E. coli*.

Sequestration of the nanoparticles by the EPS matrix was demonstrated by TEM analysis. Figure 4 shows TEM images of *E. coli* exposed to ZnONPs (A) and SiO_2_NPs (B) for 16 h, with nanoparticles entrapped on the cell surface. Deposition of ZnONPs into the EPS matrix indicates that they can combine with the EPS to form a flocculating constituent that accumulates on the surface of the *E. coli* cell, appearing as clear clusters (Fig. 4A). The amplified image in the bottom-left portion of Fig. 4A shows that ZnONPs entrapped within the EPS matrix form ZnONPs-EPS flocs (also called NP inclusions). The SiO_2_NPs exhibit a completely different deposition pattern (Fig. 4B), where the particles are uniformly dispersed on the bacterial surface, but flocs are not evident. The amplified image in the top-left portion of Fig. 4B shows that the dispersed SiO_2_NP layer is ~56 nm thick. This Zn and Si signals of EDS analysis for these regions (Figure S3 of Supplementary Information) reconfirm the ZnONPs/SiO_2_NPs entrapped within EPS matrix. These results indicate that the bacterial EPS plays a critical role in sequestering NPs and mitigating NP stress. In addition, our previous study confirmed that metal ions with high oxidation states, such as Ag^+^, can be reduced into silver nanoparticles^18^. In the present study, this possibility can be ruled out by the consistency of diffraction patterns of ZnONPs before and after reaction with *E. coli* (Figures S4C and S5 of Supplementary Information). This reconfirmed that NPs in EPS matrix originated from the added NPs, not reduction product of dissolving Zn^2+^.

### Mechanisms of NP sequestration by bacterial EPS

**Figure 5:**
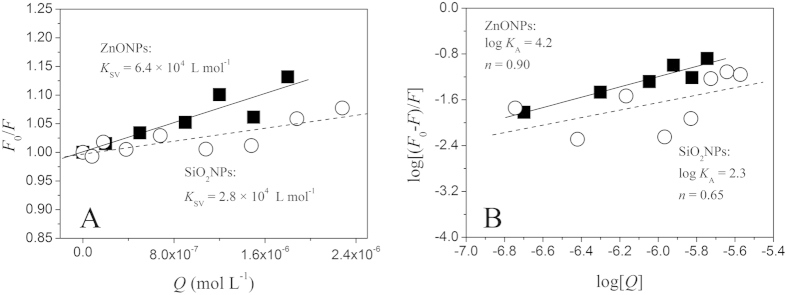
Fluorescence quenching of tryptophan-related chromophores in EPS caused by ZnONPs and SiO_2_NPs. (**A**) Stern-Volmer plot; (**B**) plot of log [(*F*_0_−*F*)/*F*] vs. log [*Q*]. This result was obtained via the aforementioned micro-titration experiments (see Materials and Methods). The changes in the fluorescence intensity of tryptophan-related chromophores in the EPS located at EX/EM = 278 nm/339 nm were recorded during the ZnONP/SiO_2_NP titration. The statistical parameters are A: ZnONPs, *R*^2^ = 0.91, p < 0.01; SiO_2_NPs: *R*^2^ = 0.83, p < 0.01; B: ZnONPs, *R*^2^ = 0.95, p < 0.01; SiO_2_NPs: *R*^2^ = 0.58, p = 0.14.

**Figure 6:**
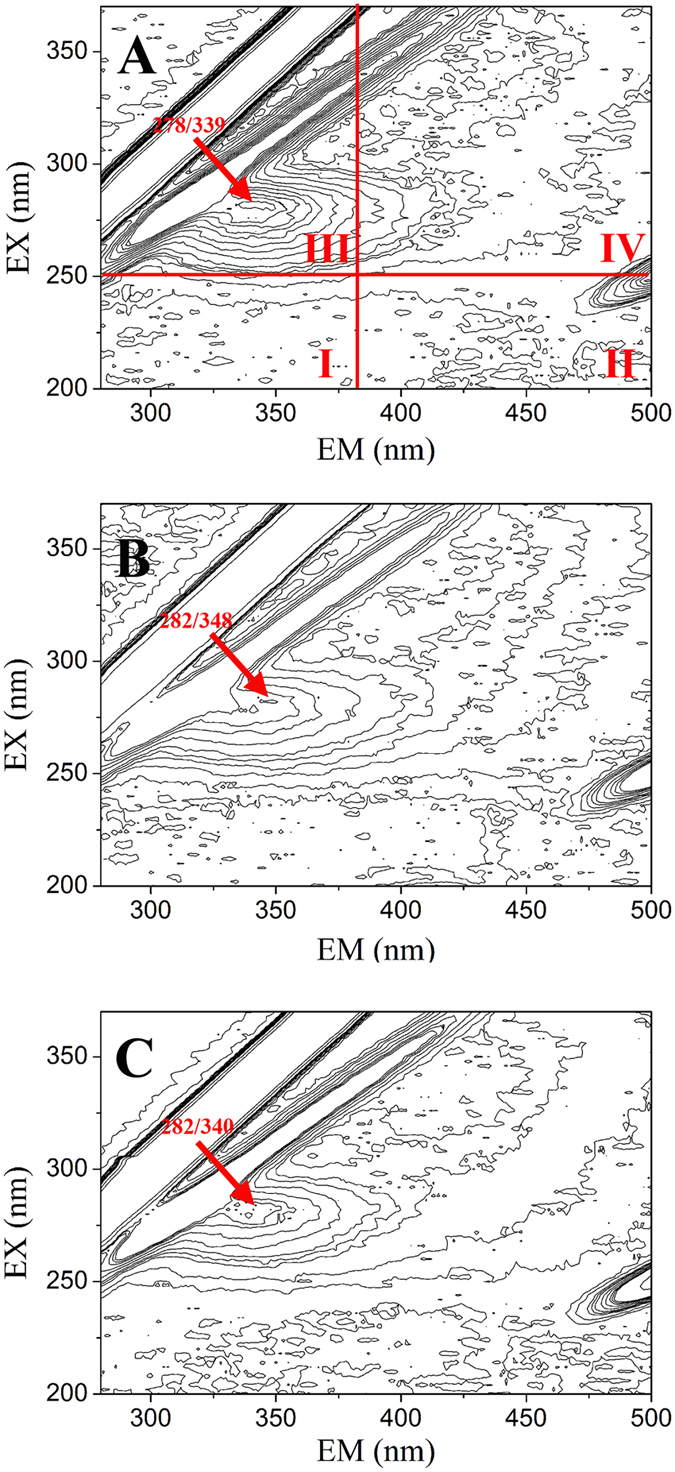
Location of three-dimensional fluorescence peaks for the EPS extracted sample (**A**) and for EPS titrated by ZnONPs (**B**) and SiO_2_NPs (**C**). In Fig. 3A, region I is biogenic aromatic proteins containing tyrosine, tryptophan and complexed tryptophan and protein-like compounds (EX = 200–250 nm, EM = 280–380 nm); region II represents fulvic acid-like compounds (EX: 210–250 nm, EM = 380–425 nm); region III represents aromatic proteins and soluble microbial by-product-like proteins; region IV contains humic acid-like structures. After titration, the EEM spectra shifted from the initial EX_278 nm_/EM_339 nm_ (**A**) to EX_282 nm_/EM_348 nm_ (**B**) and EX_282 nm_/EM_340 nm_ (**C**), respectively, indicating that major protein-like structures in EPS were involved in complexation with ZnONPs/SiO_2_NPs.

On the basis of changes in the fluorescence spectra and intensity of the EPS caused by NP-EPS sequestration, the diffusion-controlled or chemically static quenching of the EPS by NPs can be discriminated on the basis of equation 2 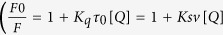, see Materials and Methods). Figure 5A shows the Stern-Volmer plots of *F*_0_/*F* versus [ZnONPs] and [SiO_2_NPs] at pH 7.0 and 25 °С. Linear fitting of the data resulted in calculated *K*_SV_ values of 6.4 × 10^4^ L mol^−1^ and 2.8 × 10^4^ L mol^−1^ for the ZnONPs and SiO_2_NPs, respectively. For tryptophan-related chromophores, *τ*_0_ is known to be ~5 × 10^−9^ s. Thus, from equation 2, the *K*q values were calculated to be 1.28 × 10^13^ and 5.6 × 10^12^ L mol^−1^ s^−1^ for ZnONP- and SiO_2_NP-formed bioconjugates, respectively. In general, the maximum *K*_q_ value for a diffusion-controlled quenching process involving a biopolymer is approximately 2.0 × 10^10^ L mol^−1^ 24. The higher quenching rate constants imply a static quenching process related to the formation of EPS-NP complexes^25^. The assumption of this static quenching process is reconfirmed by the red-shift of the three-dimensional fluorescence spectra of EPS corresponding to the maximum absorption peak after reaction with NPs (Fig. 6). The peaks of the natural tryptophan probe related to protein-like structures (amide II) or/and the glycoprotein of EPS were located at 278 nm/338 nm (excitation/emission). After reaction with ZnONPs and SiO_2_NPs, these peaks shifted to 282 nm/348 nm and 282 nm/340 nm, respectively. Because the diffusion-controlled dynamic quenching processes cannot cause such significant wavelength shifts^26^, these observed shifts indicate that the EPS matrix sequesters the ZnONPs and SiO_2_NPs through the formation of NP-EPS complexes.

The static quenching process implies that EPS sequestrates the SiO_2_NPs and ZnONPs through the formation of a non-fluorescent fluorophore-free hydrated metal ion/soluble labile metal complex at the aqueous EPS-NP interface^27^,^28^. Figure 5B shows that the log[(*F*_0_-*F*)/*F*] versus log[*Q*] plot is linear (Fig. 5B) (eq. 3). From the slope of the linear fitting plots (Fig. 5B), the binding site numbers, *n*, were determined to be 0.90 (ZnONPs) and 0.65 (SiO_2_NPs). These *n* values are approximately equal to one, indicating there a single binding site exists for the ZnONP/SiO_2_NP- tryptophan combination. From the intercept (log*K*_A_), the binding constant, *K*_A_, was calculated to be 1.6 × 10^4^ and 2.0 × 10^2^ L mol^−1^, respectively, indicating that EPS has a stronger capacity to sequester ZnONPs than to sequester SiO_2_NPs. This difference is explained by the measured zeta potentials. Apparently, ZnONPs with positive charges (20.03 ± 1.49 mV) are easily sequestered by electronegative EPS compared to SiO_2_NPs (−21.17 ± 2.9 mV). In addition, the SiO_2_NP-EPS combination in Fig. 5B exhibits a poor linear correlation (*R*^2^ = 0.58), indicating that the weak interaction between SiO_2_NPs and EPS via major binding sites is easily affected by other interaction forces, such as electrostatic forces.

**Figure 7:**
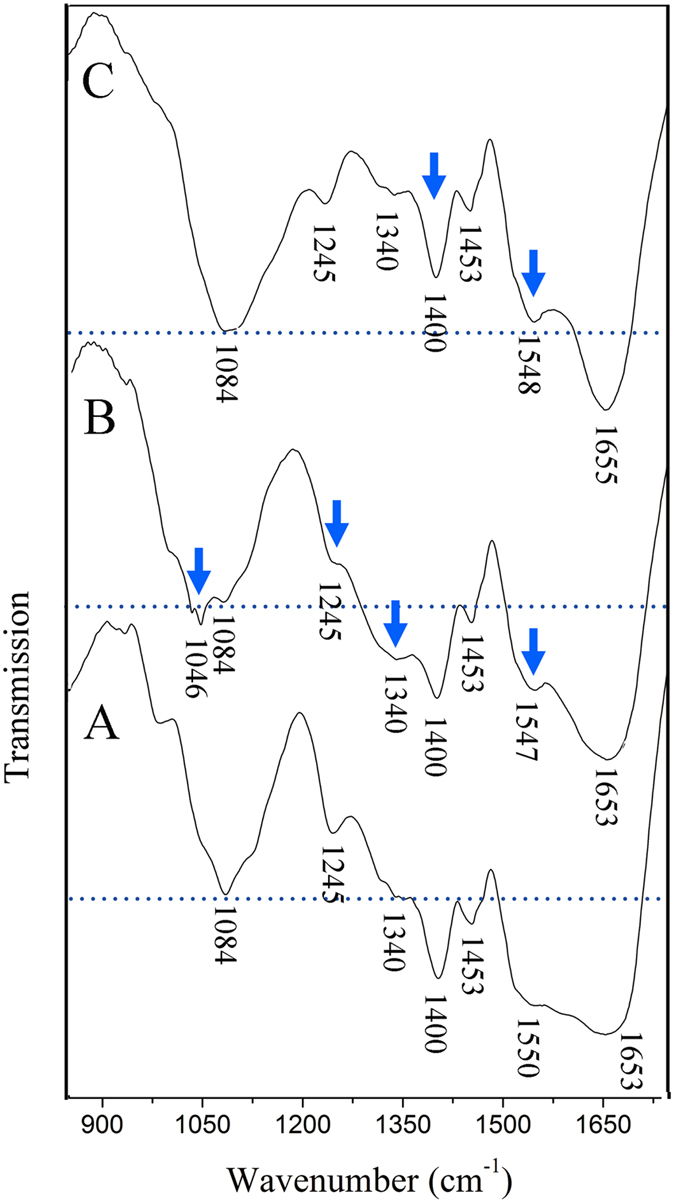
Comparison of FTIR spectra of EPS before and after reaction with NPs. (**A**) Pristine EPS. (**B**) EPS reacted with ZnONPs. (**C**) EPS reacted with SiO_2_NPs. Baselines (dashed) were set according to the stretching band of the stable polysaccharide hydroxyl to compare the changes of other groups.

Figure 7 shows the changes in the organic moieties and functional groups of the EPS by comparison of its FTIR spectra before and after reaction with ZnONPs and SiO_2_NPs. For the pristine EPS (Fig. 7A), the band at 1,653 cm^−1^ is ascribed to C=O stretching (amide I), whereas the band at 1,550 cm^−1^ is ascribed to the N-H bending and C-N stretching (amide II) in peptides^29^,^30^. The band at 1,453 cm^−1^ is attributed to the deformation vibration of CH_2_^29^,^31^. The bands at 1,400 and 1,340 cm^−1^ are related to the stretching of C−O in carboxylates^12^ and N-H stretching in peptides^32^, respectively, whereas the band near 1,245 cm^−1^ is related to the deformation vibration of C=O in carboxylic acids^29^. The bands near 1084 cm^−1^ are assigned to carbohydrate backbones^33^. The band at 1,046 cm^−1^ is caused by the stretching vibration of C-O-C in sugar derivatives^34^. These results suggest that the EPS contains mainly protein, saccharides, and carboxylates. After reaction with ZnONPs (Fig. 7B), the bands of N-H/C-N (amide II) in peptides (1,340 cm^−1^ and 1,550 cm^−1^) and C=O in carboxylic acids (1,245 cm^−1^) become much weaker, indicating that, in addition to the major protein-like structures (amide II) or/and glycoprotein of the EPS in Fig. 5, carboxylate-like structures (C=O) in the EPS are also involved in complexation with ZnONPs at other subordinate binding sites (Because content of carboxylic acids is very low, only 3.6 mg g^−1^, chromophore-producing fluorescence for carboxylate-like structures cannot be observed from Fig. 6). Additionally, the band corresponding to the C-O-C group of sugar derivatives (1046 cm^−1^) becomes much sharper and stronger (Fig. 7B), implying the possibility of trivial reducing sugar adsorbing these nanoparticles^14^. Furthermore, after reaction with SiO_2_NPs (Fig. 7C), the N-H and C-N (amide II) bands of the peptides and the C=O bands of the carboxylic acids (1245 cm^−1^) become much weaker compared to the baseline (1084 cm^−1^). This decrease in intensity suggests that the sequestration of negative SiO_2_NPs is also attributable to mainly protein-like amphoteric polymers (N-H and C-N in amide II) rather than carbonyl groups (C=O), similar to ZnONP sequestration. Taken together, these observations suggest that the EPS on the surface of bacterial cells plays a critical role in retarding the particle-specific bactericidal activity of NPs. Given the amount and composition of the EPS produced by bacteria, the bacterial EPS has important implications for the bactericidal efficacy and environmental impact of the NPs.

## Methods

### Materials

Dry ZnONPs and SiO_2_NPs (particle sizes of 15 nm, analytical reagents of 99.5%) with a low ionic toxicity were purchased from Nanjing Emperor Nano Material Co., Ltd., China. These NPs were preliminarily analyzed in our experimental laboratory. The test results were in line with the nominal particle diameters (Figure S1A and B of the Supplementary Information). The ZnONPs appeared an ellipsoidal and polycrystalline structure (Figure S4A and C of the Supplementary Information); and SiO_2_NPs showed an ellipsoidal and amorphous structure (Figure S4B and D of the Supplementary Information). The zeta potentials were 20.03 ± 1.49 mV for the ZnONPs and −21.17 ± 2.90 mV for the SiO_2_NPs at pH 7.0, 25 °C, and KNO_3_ electrolyte (1 mmol L^−1^, no *E .coli* cells). Biotechnology-grade peptone and yeast extract were purchased from Oxoid Co., Ltd., Basingstoke, Hampshire, England. Sodium chloride (NaCl, 99.0%), zinc chloride (ZnCl_2_, 99%) and sodium metasilicate (Na_2_SiO_3_·9H_2_O, 98%) were purchased from Sigma-Aldrich (St. Louis, MO, USA). Ultrapure water produced by a Milli-Q gradient system (electrical conductivity 18.2 MΩ·cm, Millipore, Bedford, MA) was used to perform all experiments.

### *E. coli* culture and removal of EPS

*Escherichia coli* (DH5a) was initially cultured in 20 mL of Luria-Bertani (LB) medium (NaCl 10 g, peptone 10 g, yeast extract 5 g, ultrapure water, 1000 mL, pH 7.5) at 37 °C for 12 h and then transferred to 1 L of fresh LB medium and cultured for another 48 h to reach the stable growth phase (see the cell growth curve in Figure S6 of the Supplementary Information). *E. coli* cells were separated from the LB medium by centrifugation (6000 *g* at 4 °C) and subsequently washed with Milli-Q water until the ultraviolet (UV) absorbance (280 nm) of the supernatant was constant (OD_280 nm_ ≈ 0.02). The bacteria were then suspended in 50% of the original volume (~1.3 × 10^8^ cell mL^−1^). The EPS was separated from the bacterial cells using a previously described method^18^,^35^, with a slight modification. The suspension was first subjected to ultrasound at an intensity of 2.7 W·cm^2^ at a frequency of 40 kHz at 4 °C for 10 min to separate the EPS from *E. coli* cells and then centrifuged for 20 min at 12,000 *g* and 4 °C to pellet the *E. coli* cells, while leaving the dissolved EPS in suspension. The supernatant was collected and filtered through a 0.22-μm membrane (Anpel) to remove the remaining *E. coli*. The *E. coli* pellets were re-suspended in Millipore water (1 L) and stored at 4 °C as *E. coli* of removed EPS (low EPS). The extracted aqueous EPS in suspension was freeze-dried at −65 °C and stored at −30 °C for later batch reaction experiments. The total organic carbon (TOC) content (10.2 mg L^−1^) of the EPS solution was measured using a TOC-5000A analyser (Shimadzu, Kyoto, Japan). The dry weight of the EPS (after being dried at 105 °C for 24 h) was 23.5 mg L^−1^. The content of protein (97.3 mg g^−1^) in the EPS was measured by the Lowry method using bovine serum albumin as the standard^36^. The content of humic acid (3.6 mg g^−1^) was measured by the modified Lowry method using commercial humic acid (Fluka) as the standard^37^. The carbohydrate content (309.4 mg g^−1^) was determined by the phenol-sulphuric acid method using glucose as the standard^38^. The DNA content (0.5 mg g^−1^) was measured by the diphenylamine colorimetric method using calf thymus DNA as the standard^39^. The obtained DNA content was low, in agreement with a previous report of negligible cell lysis during EPS extraction^40^.

### Effect of EPS on *E. coli* survival under NP stress

The effect of EPS on *E. coli* survival was investigated in the presence of ZnONPs and SiO_2_NPs at 37 °C. The dose-response relationships were determined under the following four test conditions: pristine (pristine *E. coli*); low EPS (EPS removal by sonication/centrifugation); pristine-NP dialysis (pristine *E. coli* with NP dialysis); and low EPS-NP dialysis (EPS removal with NP dialysis). The NP dialysis isolation treatment was used to investigate the particle-specific toxic effects of ZnONPs/SiO_2_NPs (pristine-NP dialysis and low EPS-NP dialysis). Here, the NPs were separated from *E. coli* cells using a dialysis membrane (3,500 Da), such that the released soluble zinc and silicon could permeate the membrane but the NPs themselves could not. The effect of the dialysis membrane on the NPs and dissolved ions was ruled out by a sorption experiment because the adsorbing capacity of NPs/ions by the dialysis membrane (3-cm length) was less than 0.5% of the added NPs/ions (Figure S7 of Supplementary Information). In brief, LB medium (36 mL) was added to a 100-mL glass conical flask equipped with a permeable silica-gel stopper, followed by the addition of aqueous stock solutions of 20 g L^−1^ NPs (ZnONPs or SiO_2_NPs,) and *E. coli* solution (4 mL) to the desired concentrations (0−500 mg L^−1^ of ZnONPs or SiO_2_NPs, 1.3 × 10^7^ cell mL^−1^ of *E. coli*). For the dialysis experiments, the NP stock solutions were added into dialysis bags with a molecular weight cut-off of 3,500 (3-cm length). LB medium was added to the dialysis bags to the desired 4 mL, and the bags were sealed using PTFE string. The total volume for each reaction solution, including the dialysis and non-dialysis treatments, was 40 mL. The effect of dialysis membrane on NPs and ions (Zn^2+^and SiO_3_^2−^) were rule out by an adsorption test (Figure S7 of Supplementary Information). The volume ratio of NP stock solution, if added, was maintained at less than 2.5% for the reaction solution. *E. coli* survival was monitored under the aforementioned four different EPS conditions (pristine-NPs, low-EPS-NPs, pristine-NP dialysis, and low-EPS-NP dialysis).

All samples were incubated in the dark with horizontal vibration at 160 revolutions per minute (rpm) and at 37 ± 0.5 °C. In such speed (without any *E. coli*), the NPs can suspend in solutions. If the *E. coli* was added to NP solution, the aggregation will occur rapidly due to NP-*E. coli* combination. *E. coli* cells in LB medium were cultured for 16 h until the exponential growth phase (Figure S6 of the Supplementary information) and were harvested by centrifugation (6,000 *g* at 4 °C for 10 min)^41^. After removal of the supernatant, the cell pellets were re-suspended with Milli-Q water and *E. coli* survival was assessed by viable plate counts after 10^6^–10^8^-fold gradient dilution^11^. In brief, 50 μL of the diluted suspensions were spread onto LB solid plates (1 L of LB medium plus 15 g of agar powder) and cultured for 20 h at 37 °C. On the basis of the counted colonies, the *E. coli* survival rate was calculated as the ratio of the *E. coli* density in the presence (*A*) versus absence (*B*) of NPs:
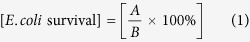


### Release of soluble zinc/silicon from NPs

The soluble zinc and silicon released by the ZnONPs/SiO_2_NPs were determined to explore the effect of NP dissolution on *E. coli* survival with and without the EPS. After the reaction of the NPs with *E. coli*, the released soluble zinc and silicon were collected by a filtration method^11^. The solutions were first filtered through a 0.22-μm membrane. The *E. coli* and NPs on the membrane were re-suspended by adding Milli-Q water to wash the *E. coli*/NP-adsorbed ions, followed by repeated washing/filtration until the soluble zinc and silicon in the supernatant could not be detected. The released soluble zinc and silicon were determined by inductively coupled plasma-atomic emission spectrometry (ICP-AES); the instrument was calibrated using aqueous zinc chloride (ZnCl_2_) and sodium metasilicate (Na_2_SiO_3_·9H_2_O) solutions.

In addition, on the basis of the soluble zinc/silicon concentrations, equivalent concentrations of Zn^2+^and SiO_3_^2−^ ions were prepared to explore the effect of soluble zinc/silicon ions on *E. coli* growth and to further rule out ion-specific bactericidal activity. The experimental procedure was identical to that previously described for the effect of the EPS on *E. coli* survival under NP stress, with the exception that the ZnONPs/SiO_2_NPs were replaced by Zn^2+^ and SiO_3_^2−^, respectively.

### Fluorescence quenching with micro-titration

Three-dimensional excitation-emission matrix (EEM) fluorescence spectroscopy combined with micro-titration (EEM-QM) was used to explore the interaction between NPs and extracted EPS, as described previously^42^. The ZnONP or SiO_2_NP stock solutions (10 mmol·L^−1^) were gradually titrated into the 20-mL aqueous EPS solutions (0.5 mg L^−1^, dry weight basis) using a chromatographic injector with a scale of 50 μL, followed by magnetic stirring for 20 min at 160 rpm, pH 7.0 and 25 °С. The fluorescence spectra and corresponding intensities were recorded at an excitation wavelength of 200–380 nm and an emission wavelength of 250–500 nm (fluorescence wavelength resolution of ≥1 nm) (F96PRO, LengGuang). The NP stock solution was then titrated into the aqueous EPS solution, and the fluorescence spectra and intensities were recorded again after reaction for 20 min at 160 rpm and 25 °С. The procedures were repeated until no significant change in the fluorescence intensity was observed. In addition, a separate study was performed to explore the effect of NP dissolution on the fluorescence quenching of the EPS. Equivalent Zn^2+^ and SiO_3_^2−^ concentrations were used to titrate the EPS (0.5 mg L^−1^, dry weight basis). No marked fluorescence quenching by Zn^2+^ or SiO_3_^2−^ was detected (Figure S8), indicating that the fluorescence quenching of the EPS could be attributed to the NPs, not to dissolved zinc or silicon. The possibility of fluorescence quenching caused by the dissolution media of the NPs was ruled out during the titration.

The EPS-NP combination could be well described on the basis of the relationship between the fluorescence intensity and the quencher concentration^43^ (ZnONPs/SiO_2_NPs). The Stern-Volmer equation is given by^24^
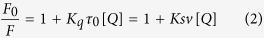
where *F*_0_ and *F* are the relative fluorescence intensities of the EPS in the absence and presence of the quencher (ZnONPs or SiO_2_NPs), respectively, *K*_q_ is the bimolecular quenching rate constant, *τ*_0_ is the average lifetime of the fluorophore in the absence of the quencher, and [*Q*] is the concentration of the quencher. *K*_SV_ is the Stern-Volmer quenching constant, which represents the efficiency of quenching. For the static quenching process, the following equation is used to determinate the binding constant or association constant (*K*_A_) and binding sites (*n*)^44^:
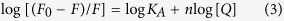


### TEM and FTIR analyses

Transmission electron microscopy (TEM) and selected area electron diffraction (SAED) analysis was applied to characterize the sequestration of NPs in the EPS matrix on the surface of *E. coli*. After being mixed for 16 h at 37 °C in the dark, the *E. coli* samples originally containing NPs were centrifuged for 10 min at 4 °C and 6,000 *g*. The cell/NP samples were re-suspended, washed (Milli-Q water), and centrifuged three times to remove the LB medium. One portion of the cell/NP pellet was placed onto a carbon-coated copper grid for TEM imaging using a bright-field detector on a JEM-2100 (JEOL, Japan). To identify the structural components of the EPS responsible for entrapping the NPs, FTIR analysis was performed to characterize the chemical structures of the EPS before and after reaction with the NPs for 16 h. The FTIR spectra of the freeze-dried EPS mixed with KBr (mass ratio of 1:100) were acquired on a Nicolet NEXUS870 (Thermo Scientific, USA).

## Additional Information

**How to cite this article**: Wang, Q. *et al*. Sequestration of nanoparticles by an EPS matrix reduces the particle-specific bactericidal activity. *Sci. Rep.* **6**, 21379; doi: 10.1038/srep21379 (2016).

## Supplementary Material

Supplementary Information
