# Supplementary material for: Sequestration of nanoparticles by an EPS matrix reduces the particle-specific bactericidal activity
Source: Sci Rep. 2016 Feb 9;6:21379. doi: 10.1038/srep21379 (PMC4809067; doi:10.1038/srep21379)
Supplement: Supplementary Information [file srep21379-s1.pdf]

Supplementary information:

Sequestration of nanoparticles by an EPS matrix reduces the particle-specific bactericidal activity

Qian Wang[1,3], Fuxing Kang[2,*], Yanzheng Gao[2,*] Xuewei Mao[2], Xiaojie Hu[2]

[1] State Key Laboratory of Lake Science and Environment, Nanjing Institute of Geography and Limnology, Chinese Academy of Sciences, Jiangsu 210008, China

[2] Institute of Organic Contaminant Control and Soil Remediation/College of Resources and Environmental Sciences, Nanjing Agricultural University, Jiangsu 210095, China

[3] School of Earth Sciences and Engineering, Nanjing University, Jiangsu 210046, China

*Correspondence and requests for materials should be addressed to F.X.K (kangfuxing@126.com) and Y.Z.G. (gaoyanzheng@njau. edu.cn)

Manuscript prepared for *Scientific Reports*

October 27, 2015

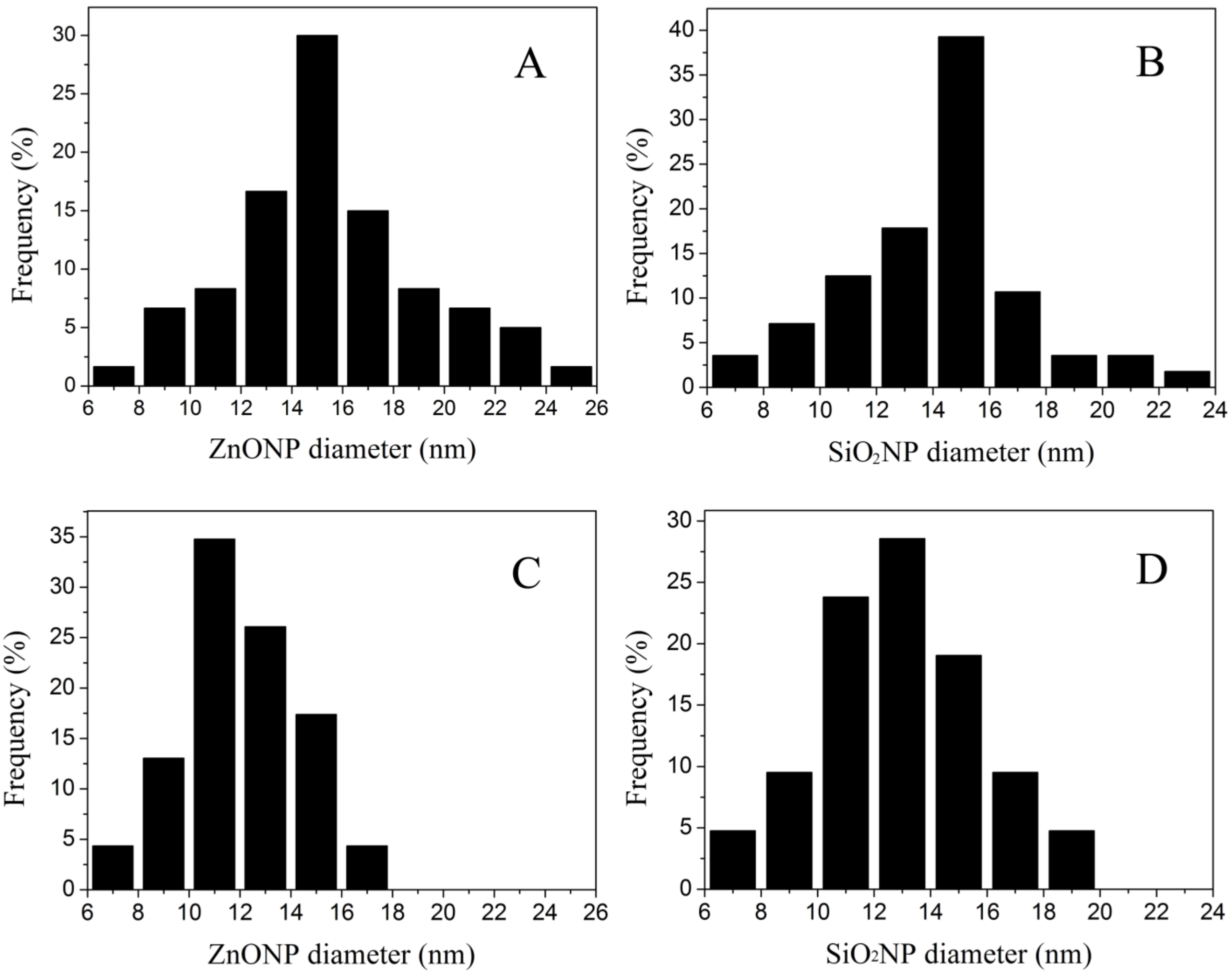

Figure | S1 Diameter of ZnONPs and SiO_2NPs after reaction of with *E. coli*. (A): Pristine ZnONPs; (B): SiO_2NPs; (C): ZnONPs after reaction with *E. coli*; (D): SiO_2NPs after reaction with *E. coli*. The data were calculated on the basis of Figure 4A and B in text and Figure S3A and B of the Supplementary Information.

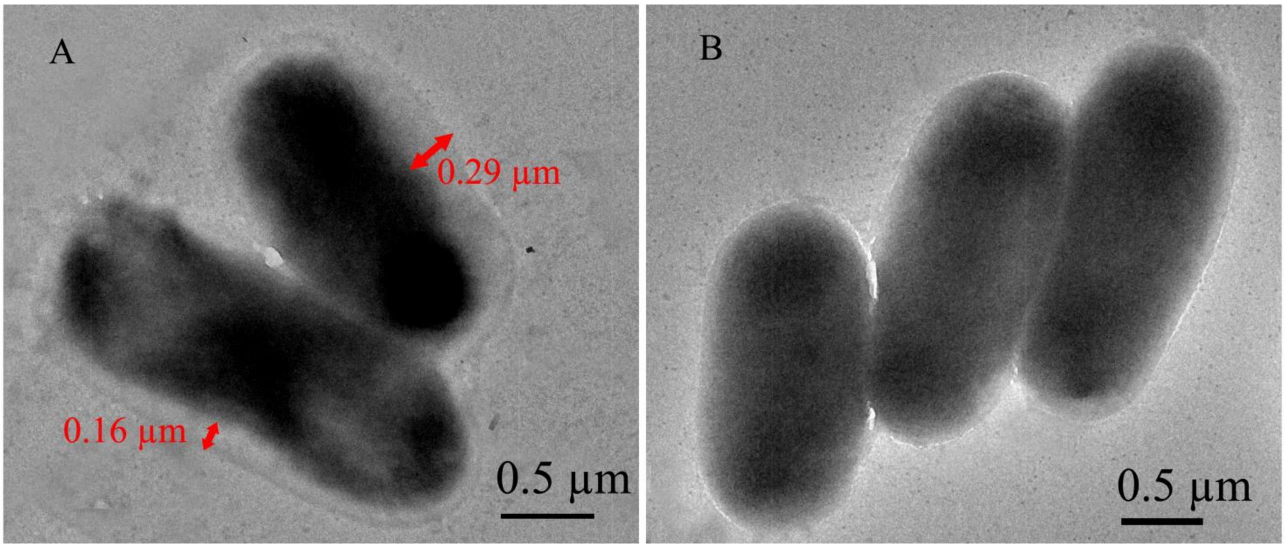

Figure | S2 EPS permeable barrier on *E. coli* surface before (A) and after EPS manipulation (B). Red arrows present the thickness of EPS permeable barrier.

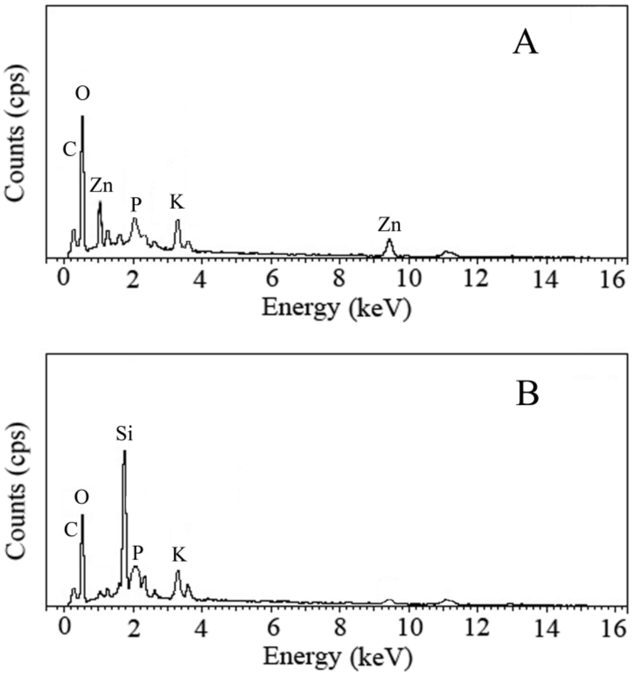

Figure | S3 Energy dispersive spectroscopy(EDS) analysis of ZnONPs (A) and SiO_2NPs (B) on surface of *E. coli*. The C, P, and K signals in the EDS images are from the EPS on surface of *E. coli*.

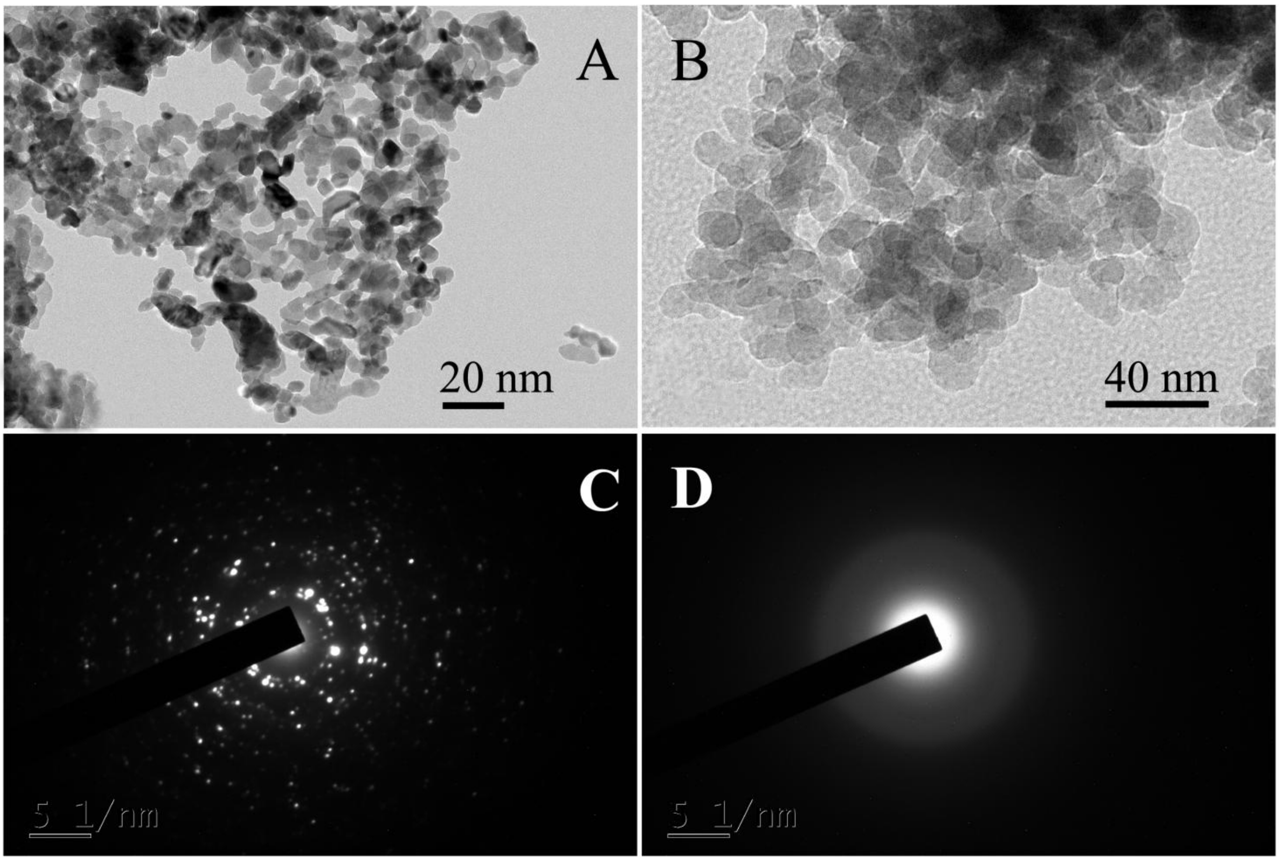

Figure | S4 TEM and SAED analysis of pristine ZnONPs (A and C) and SiO_2NPs (B and D).

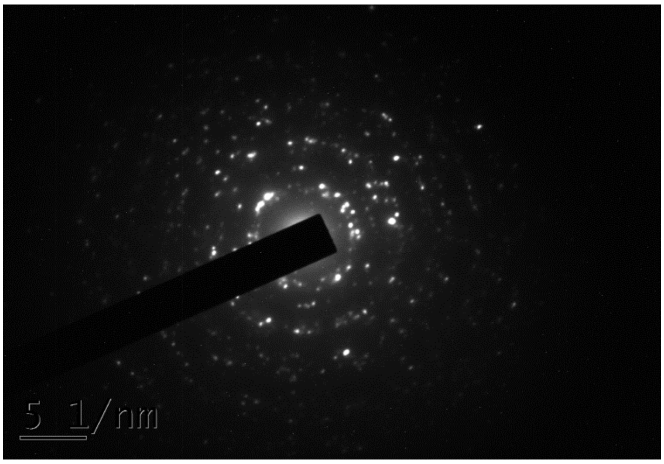

Figure | S5 SAED image of ZnONPs on *E. coli* surface.

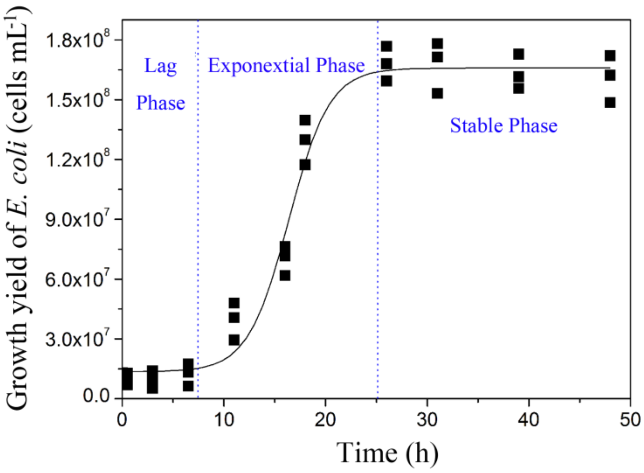

Figure | S6 The growth curve of *E. coli* in LB medium for 48 h at 37 ºC.

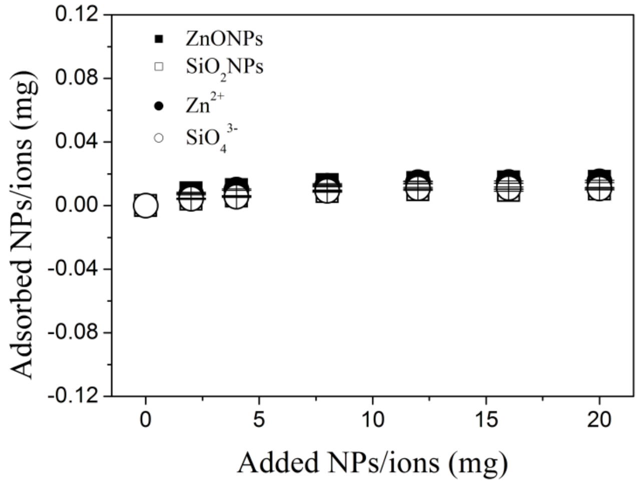

Figure | S7 Negligible effect of dialysis membrane on NPs and ions. The reaction solutions were 40 mL. The dialysis bag with a with molecular weight cut off 3500 (3-cm length) was used to act as the adsorbent. The aqueous NP and ion solutions (40 mL) were used in this sorption experiment, and no LB medium was used. The batch experiments were performed at 37 ℃ for 16 h.

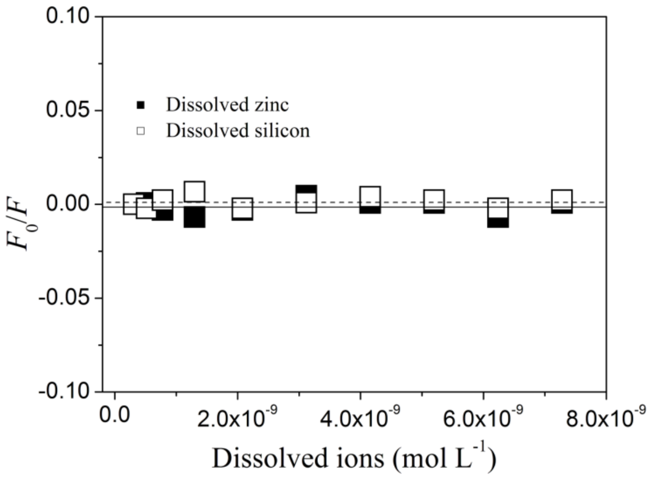

Figure | S8 Negligible fluorescence quenching resulted from ZnONP and SiO_2NP dissolution. Basing on slight dissolution of NPs, equivalent Zn^{2+} and SiO_3^{2-} ions were used to titrate the EPS (0.5 mg L^{-1}, dry weight basis), it was not observed the obvious fluorescence quenching. This indicated that fluorescence quenching of EPS during the titration could be attributed to NP, rather than dissolving zinc or silicon.
